# Cytoplasmic RAP1 mediates cisplatin resistance of non-small cell lung cancer

**DOI:** 10.1038/cddis.2017.210

**Published:** 2017-05-18

**Authors:** Lu Xiao, Xiaoying Lan, Xianping Shi, Kai Zhao, Dongrui Wang, Xuejun Wang, Faqian Li, Hongbiao Huang, Jinbao Liu

**Affiliations:** 1 Key Laboratory of Protein Modification and Degradation, SKLRD, Affiliated Cancer Hospital and School of Basic Medical Sciences, Guangzhou Medical University, Guangzhou, Guangdong 511436, China; 2 Department of Hematology and Hematopoietic Cell Transplantation, City of Hope, Duarte, CA 91010, USA; 3 Division of Basic Biomedical Sciences, Sanford School of Medicine of the University of South Dakota, Vermillion, SD 57069, USA; 4 Department of Laboratory Medicine and Pathology, University of Minnesota, Dwan Variety Club Cardiovascular Research Center, Minneapolis, MN 55455, USA

## Abstract

Cytotoxic chemotherapy agents (e.g., cisplatin) are the first-line drugs to treat non-small cell lung cancer (NSCLC) but NSCLC develops resistance to the agent, limiting therapeutic efficacy. Despite many approaches to identifying the underlying mechanism for cisplatin resistance, there remains a lack of effective targets in the population that resist cisplatin treatment. In this study, we sought to investigate the role of cytoplasmic RAP1, a previously identified positive regulator of NF-*κ*B signaling, in the development of cisplatin resistance in NSCLC cells. We found that the expression of cytoplasmic RAP1 was significantly higher in high-grade NSCLC tissues than in low-grade NSCLC; compared with a normal pulmonary epithelial cell line, the A549 NSCLC cells exhibited more cytoplasmic RAP1 expression as well as increased NF-*κ*B activity; cisplatin treatment resulted in a further increase of cytoplasmic RAP1 in A549 cells; overexpression of RAP1 desensitized the A549 cells to cisplatin, and conversely, RAP1 depletion in the NSCLC cells reduced their proliferation and increased their sensitivity to cisplatin, indicating that RAP1 is required for cell growth and has a key mediating role in the development of cisplatin resistance in NSCLC cells. The RAP1-mediated cisplatin resistance was associated with the activation of NF-*κ*B signaling and the upregulation of the antiapoptosis factor BCL-2. Intriguingly, in the small portion of RAP1-depleted cells that survived cisplatin treatment, no induction of NF-*κ*B activity and BCL-2 expression was observed. Furthermore, in established cisplatin-resistant A549 cells, RAP1 depletion caused BCL2 depletion, caspase activation and dramatic lethality to the cells. Hence, our results demonstrate that the cytoplasmic RAP1–NF-*κ*B–BCL2 axis represents a key pathway to cisplatin resistance in NSCLC cells, identifying RAP1 as a marker and a potential therapeutic target for cisplatin resistance of NSCLC.

Lung cancer is a leading cause of tumor-related mortality around the world. Non-small-cell lung cancer (NSCLC) represents a heterogeneous group of tumors that account for >85% of the newly diagnosed lung cancer incidents.^1^ Multiple approaches have been applied for the treatment of NSCLC, among which are the apoptosis-induction chemotherapy agents such as cisplatin (CP).^1, 2^ But unfortunately, considerably high rate of relapse occurs following CP treatment despite the approaches aiming to overcome resistance.^3, 4, 5^ It is thus necessary to develop strategies that act on the CP-resistant subpopulation of NSCLC and reverse the resistance in these tumor cells.

During the past decade, great effort has been made to identify the extratelomeric functions of telomere-associated proteins.^6^ One of these factors is RAP1 (repressor/activator protein-1), also known as TRF2IP (telomeric repeat binding factor-2 interacting protein-1), which was originally identified as part of the telomere-binding complex.^7, 8^ Considered as a shelterin protein component, RAP1 has been found to associate with multiple types of human cancer, including NSCLC.^9, 10, 11, 12^ However, genetic modification studies have revealed that RAP1 is not required in the maintenance of human telomere length and telomere heterochromatic structure,^13, 14^ indicating that other mechanisms must be involved regarding the function of RAP1 in oncogenesis. Indeed, RAP1 was identified to bind with extratelomeric sites through a consensus motif, helping with gene transcriptional regulation.^15, 16^ Meanwhile, cytoplasmic RAP1 has been revealed for its essential role in modulating NF-*κ*B signaling.^17^ Specifically, the RAP1–NF-*κ*B axis was demonstrated to promote invasion of breast cancer cells.^17^ Given the broad activation of NF-*κ*B signaling in human cancer, one would infer that RAP1 might contribute to the progression of other types of tumors via a similar mechanism.

NF-*κ*B activity has been detected in both lung cancer and preneoplastic pulmonary lesions^18^ and associated with the resistance to chemotherapy.^19, 20, 21^ In lung cancer cells, independent studies have shown that cytotoxic chemotherapy using gemcitabine or TNF-*α* could induce NF-*κ*B activity and combining with NF-*κ*B inhibitor has been considered to overcome resistance.^22, 23, 24^ Further, NF-*κ*B signaling has been shown to positively regulate the expression of the apoptosis inhibitor BCL-2,^25, 26, 27, 28^ and the cancer stem-like cells, a population associated with therapy resistance, were discovered to have both high BCL-2 expression and NF-*κ*B hyperactivation in multiple types of human cancer.^29, 30^ These results suggest that BCL-2 overexpression might be, at least part of, the consequence of NF-*κ*B activation that accounts for chemotherapy resistance. More specifically, NF-*κ*B signaling was recently assessed in cervical cancer and epidermoid carcinoma cells to inhibit apoptosis and mediate CP resistance.^31, 32^ However, the mediator between chemotherapy agent and NF-*κ*B activity remains poorly understood, making it difficult to identify the target that could re-sensitize those resistant tumor cells to cytotoxic agents.

In this report, we evaluated a mediating role of cytoplasmic RAP1 in tumorigenesis of NSCLC through NF-*κ*B signaling. RAP1 is highly expressed in high-grade NSCLC samples, facilitates NF-*κ*B activation which is required for the proliferation of NSCLC cells, upregulates BCL-2 expression and mediates the resistance of NSCLC cells to CP. Meanwhile, we observed a synergistic effect between CP treatment and RAP1 deletion in tumor suppression. Our results demonstrate an extratelomeric function for RAP1 in tumor progression and suggest that RAP1 is a potential therapeutic target for CP-resistant NSCLCs.

## Results

### Expression of cytoplasmic RAP1 is associated with malignancy in NSCLC patients

**Figure 1:**
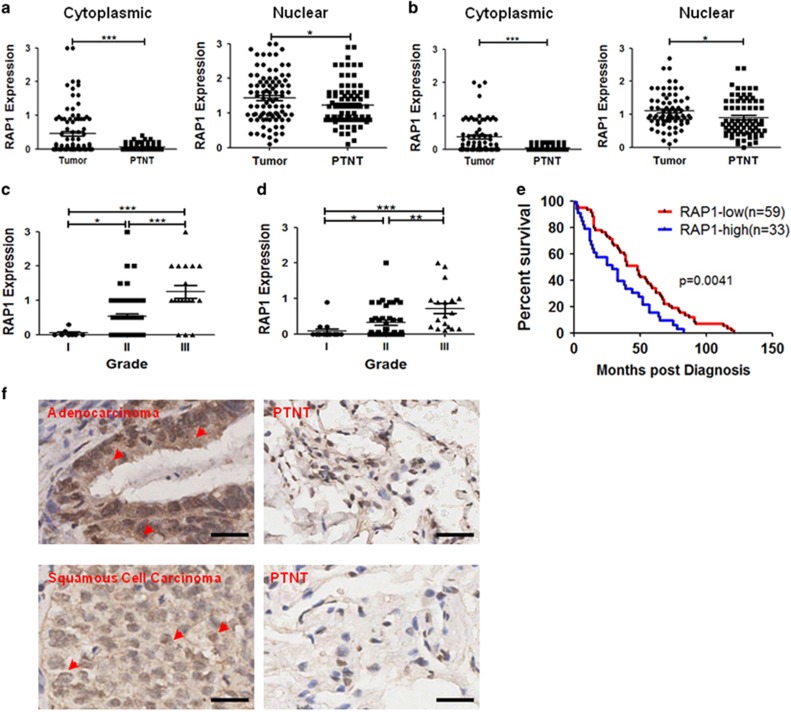
Cytoplasmic RAP1 is an indicator of high-grade NSCLC. (**a** and **b**) Expression of cytoplasmic and nuclear RAP1 in lung adenocarcinoma (**a**) and squamous cell carcinoma (**b**) tissues, as well as peri-tumoral normal tissues (PTNT). (**c** and **d**) Expression of cytoplasmic RAP1 in Grade I–III adenocarcinoma (**c**) and squamous cell carcinoma (**d**) tissues. (**e**) Kaplan–Meier analysis and Log-rank (Mantel–Cox) test of the survival between RAP1-high (*n*=33, expression >0.4) and RAP1-low (*n*=59, expression <0.4) adenocarcinoma patients. (**f**) Representative images of RAP1 expression in adenocarcinoma, squamous cell carcinoma and PTNT, arrowhead: cytoplasmic RAP1; scale bar: 10 *μ*m. *P<0.05, **P<0.01, ***P<0.001, Student’s *t*-test

It was previously reported that cytoplasmic RAP1 was detected in breast cancer tissue, and its pathology scores were positively correlated with the tumor grades.^17^ To test the hypothesis that cytoplasmic RAP1 is a biomarker for higher-grade NSCLC, we immunostained for RAP1 and quantified its cytoplasmic and nuclear expression in 93 lung adenocarcinoma and 75 lung squamous cell carcinoma tissues. The peri-tumoral normal tissue was used to reflect RAP1 expression in non-malignant cells (Supplementary Tables S1 and S2). Both cytoplasmic and nuclear RAP1 were increased in NSCLC tumors compared with normal tissues, but the difference in cytoplasmic RAP1 appears to be greater (Figures 1a, b, f, Supplementary Figures S1a and b). A higher level of cytoplasmic RAP1 expression was associated with a higher grade of NSCLCs (Figures 1c, d, Supplementary Figures S1c and d). Moreover, higher cytoplasmic RAP1 expression was associated with poorer prognosis of adenocarcinoma patients (Figure 1e; information of squamous cell carcinoma patients’ survival is unfortunately not available). These analyses identify cytoplasmic RAP1 as an indicator of high-grade NSCLC, suggesting that it may have a critical role in cancer progression.

### RAP1 is required for the growth of NSCLC cells

**Figure 2:**
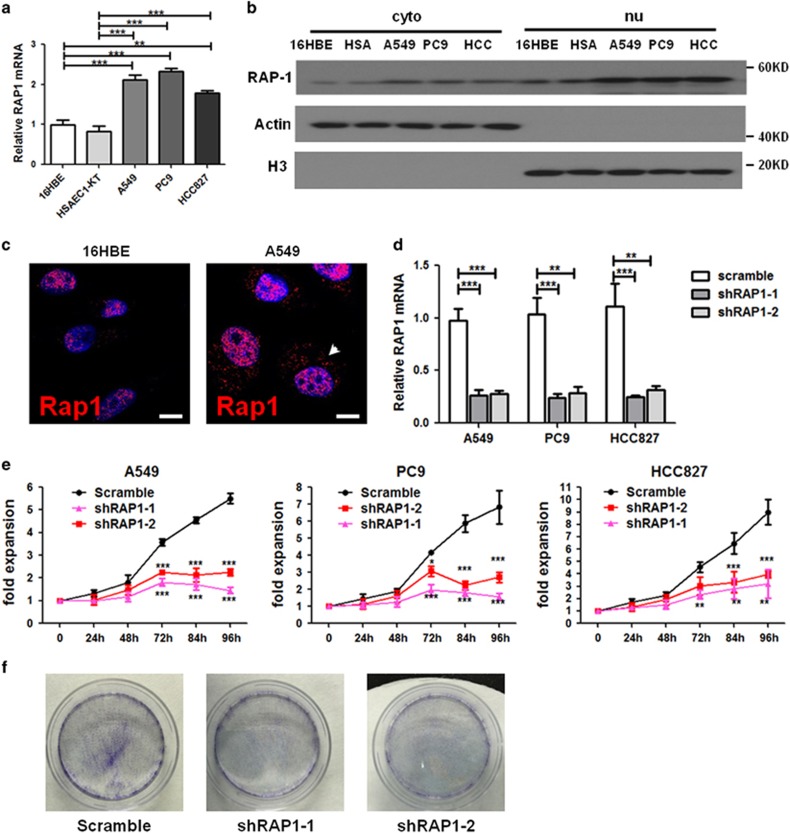
Proliferation of NSCLC cells is inhibited after Rap1 deletion. (**a**) RAP1 mRNA expression in 16HBE and HSAEC1-KT (HSA for abbreviation in following) lung epithelial cells and A549, PC9 and HCC827 NSCLC cells measured by qRT-PCR. (**b**) Western blotting analysis for RAP1 in cytoplasmic and nuclear fractions of lung epithelial and NSCLC cells, representatives of three independent experiments. (**c**) Immunofluorescence (IF) detection of RAP1 (red) in 16HBE and NSCLC cells. Arrowhead: cytoplasmic RAP1; scale bar: 5 *μ*m. (**d**) RAP1 mRNA expression in A549, PC9 and HCC827 cells after transducing lentiviral vector coding two shRNAs against RAP1 (shRAP1) or scramble shRNA. (**e**) Proliferation of NSCLC cells after transduction with shRAP1 or scramble shRNA. Cells were plated in 96-well plates, cultured for 96 h and viability determined through cell counting kit-8 (CCK-8) assay. (**f**) Colony-formation assays for A549 cells after transduction with shRAP1 or scramble shRNA, detected by Giemsa staining. Statistics were generated from three independent experiments. **P<0.01 ***P<0.001, Student’s *t*-test (two groups) or one-way analysis of variance with Bonferroni’s correction (three or more groups); error bar: ±S.D.

Although cytoplasmic RAP1 protein levels are higher in high-grade NSCLC tissues than in low-grade NSCLC tissues, it is also detected in most examined low-grade NSCLCs, prompting us to speculate that cytoplasmic RAP1 could be a signature to distinguish NSCLC cells from the normal compartment. Therefore, we compared the expression of RAP1 between three NSCLC cell lines and two lung epithelial cell lines. The NSCLC cells tested here include adenocarcinoma A549 and PC9, together with squamous cell carcinoma HCC827, while the normal cells include bronchial epithelial cells 16HBE and small airway epithelial cells HSAEC1-KT. Intriguingly, in NSCLC cells, we detected an overall higher mRNA and protein expression of RAP1, particularly in the cytoplasmic fraction compared with normal lung epithelial cells (Figures 2a and b). The increased cytoplasmic expression of RAP1 in NSCLC cells was further confirmed by immunofluorescence (Figure 2c and Supplementary Figure S2), leading to a hypothesis that RAP1 facilitates malignancy by working in the cytoplasmic compartment.

Owing to the challenge to specifically inhibit cytoplasmic RAP1, we tested the alternative hypothesis that RAP1 mediates NSCLC cell proliferation. Therefore, lentiviruses encoding either one of the two short hairpin RNA (shRNA) against RAP1 (shRAP1-1 and shRAP1-2) or a scramble shRNA were applied to three different NSCLC cells lines. RAP1 knockdown by shRAP1-1 or shRAP1-2 was evident both at the mRNA (Figure 2d) and protein levels in both cytoplasmic and nuclear fractions (Supplementary Figure S3b). RAP1-deleted NSCLC cells exhibited defect in cell viability and growth over culture (Figure 2e). Consistently, when plated at the same number, RAP1-deleted NSCLC cells formed fewer colonies after a 3-week culture (Figure 2f and Supplementary Figure S4). These results demonstrate that RAP1 is required for the growth of A549 cells.

### RAP1 induces NF-κB signaling in NSCLC cells

**Figure 3:**
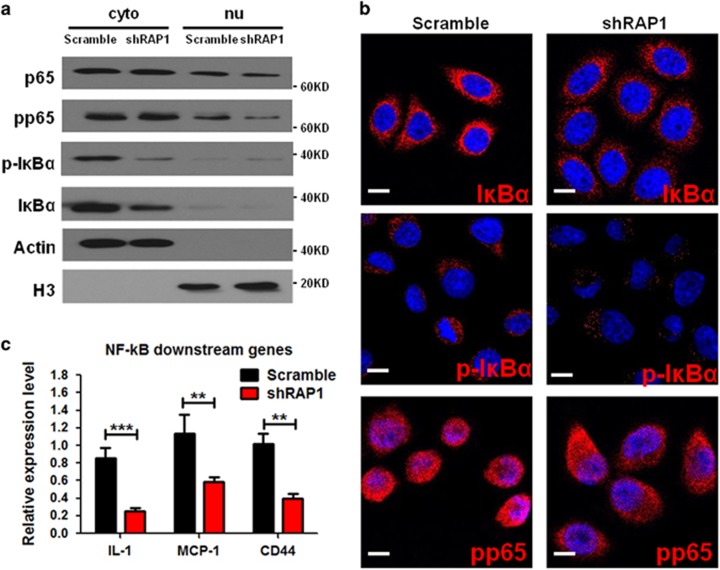
RAP1 acts through NF-*κ*B signaling to support NSCLC cell growth. (**a** and **b**) Western blotting analysis (**a**) and IF assay (**b**) of NF-*κ*B signaling factors: p65, phosphorylated-p65 (pp65), I*κ*B*α*, and phosphorylated-I*κ*B*α* (p-I*κ*B*α*) in the cytoplasmic and nuclear fractions of A549 cells transduced with shRAP1 or scramble shRNA. Scale bar: 5 *μ*m. (**c**) Expression of genes positively regulated by NF-*κ*B signaling in A549 cells transduced with shRAP1 or scramble shRNA. Statistics were generated from three independent experiments. **P<0.01, ***P<0.001, Student’s *t*-test; error bar: ±S.D.

Having proven the requirement of RAP1 in lung cancer cell proliferation, we next studied the mechanism by which RAP1 mediates cell growth. The assays described above do not attribute the function of RAP1 exclusively to the cytoplasmic compartment. However, RAP1 *per se* is insufficient for telomere protection,^13^ suggesting that nuclear RAP1 has very minor role in maintaining genomic stability in hyperproliferative tumor cells. Thus we speculated that cytoplasmic RAP1 is the main contributor to induce cell proliferation. To test this hypothesis, we detected the effect of RAP1 deletion on the activity of NF-*κ*B signaling, a known downstream effector of cytoplasmic RAP1. As was proven by previous studies, cytoplasmic RAP1 acts as IKK adaptor proteins which mediate the phosphorylation of p65.^17^ Indeed, when RAP1 was deleted in A549 cells, we observed a decrease of phosphorylated p65 in the nuclear fraction (Figures 3a and b). Phosphorylation of I*κ*B*α*, which promotes NF-*κ*B signaling, was also decreased with the deletion of RAP1 (Figure 3a and Supplementary Figure S5a). Intriguingly, transcription of I*κ*B*α* was significantly suppressed but the total I*κ*B*α* protein was only moderately decreased after RAP1 deletion (Figures 3a and b), probably due to a positive feedback of NF-*κ*B signaling. Both I*κ*B*α* and phospho-I*κ*B*α* are mainly expressed in the cytoplasmic fractions. No significant changes in IKK protein levels or in its phosphorylation were observed upon RAP1 deletion (Supplementary Figures S5b and c). To further demonstrate the inhibition of NF-*κ*B signaling, we evaluated the transcription of IL-1, MCP-1, and CD44, which are known to be positively regulated by activated NF-*κ*B. Clearly, RAP1 deletion led to the downregulation of these factors (Figure 3c). Together with the observation that RAP1 mediates NSCLC cell growth, these results suggest that the expansion of NSCLC cells is positively regulated by RAP1-mediated NF-*κ*B activation.

### RAP1 mediates CP resistance in NSCLC cells

**Figure 4:**
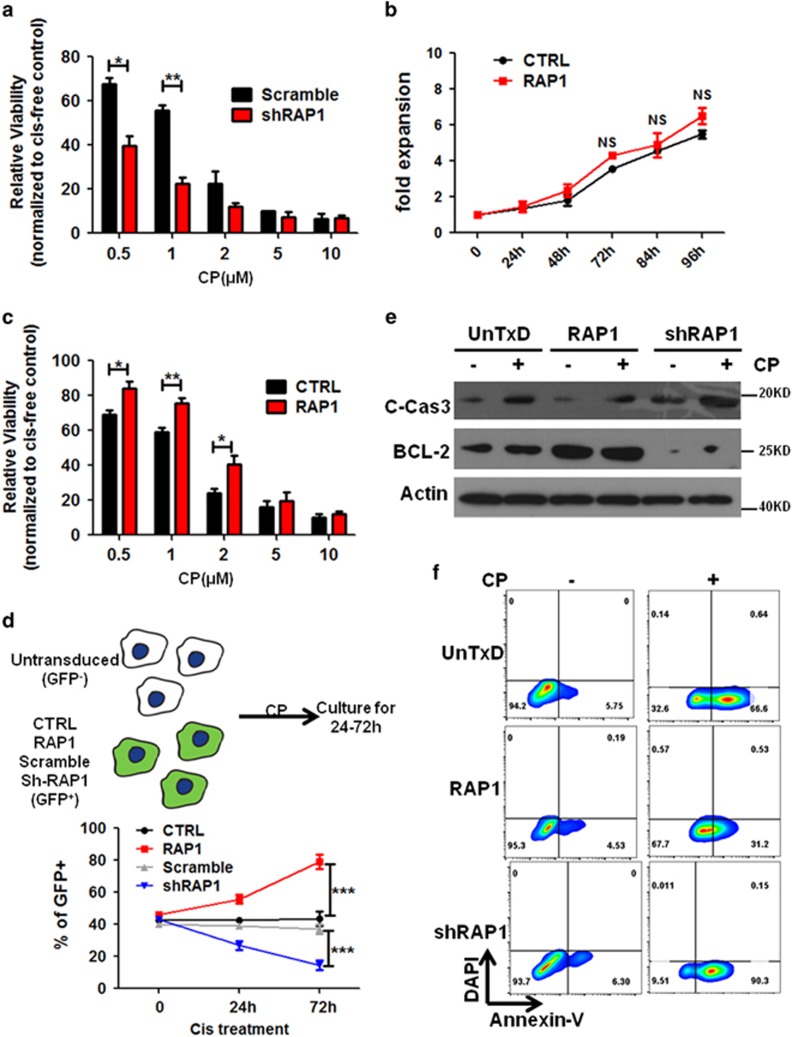
RAP1 desensitizes NSCLC cells to CP-induced cell death. (**a**) shRAP1- or scramble shRNA-transduced A549 cells were treated with different concentrations of CP for 48 h, and cell viability was determined with CCK-8 assay and normalized to the same type of cells cultured without CP. (**b**) Proliferation of A549 cells after transduction with control (CTRL) or RAP1-overexpressing (RAP1) lentiviral vectors. (**c**) Cell viability of A549 cells transduced with RAP1 or CTRL viruses and treated with different concentrations of CP for 48 h, after normalization to the same type of cells cultured in CP-free media. (**d**) Competition assay of the untransduced (GFP^−^) cells co-cultured with the cells transduced with the indicated different RAP1 modification lentiviruses (GFP^+^). Cells were treated with 2 *μ*M of CP and harvested at 24 and 72 h. Percentage of GFP^+^ cells were analyzed for the viable population determined by 4,6-diamidino-2-phenylindole staining. (**e** and **f**) Untransduced (UnTxD) A549 cells and A549 cells with RAP1 overexpression (RAP1) or RAP1 knockdown (shRAP1) were cultured in media containing 1 *μ*M CP (+) or CP-free media (−) for 24 h and analyzed for the apoptotic status with western blotting analysis for cleaved caspase-3 (C-Cas3) and BCL-2 (**e**) or with flow cytometric analysis of Annexin-V (**f**), representatives of three independent experiments. Statistics were generated from three independent experiments. *P<0.05, **P<0.01, ***P<0.001, Student’s *t*-test; error bar: ±S.D.

Activated NF-*κ*B is known to render the cancer cells resistant to chemotherapy.^21, 23^ Moreover, CD44, whose transcription is regulated through NF-*κ*B signaling, was discovered to be enriched in a CP-resistant population.^33^ Therefore, we tested the hypothesis that RAP1 suppresses the sensitivity of NSCLC cells to CP. First, the RAP1 shRNA or scramble shRNA-transduced A549 cells were treated with different doses of CP. As depicted in Figure 4a, a relative high dose of CP (>2 *μ*M) eliminated almost all cells in both groups. Nevertheless, when less CP was applied, significantly fewer RAP1-deleted cells survived the treatment (Figure 4a). Next, we investigated the effect of overexpressed RAP1 on CP sensitivity. Unexpectedly, when A549 cells were transduced to overexpress RAP1, they displayed only modest increase of growth (Figure 4b); however, a larger amount of CP was required to eliminate RAP1-overexpressed cells to an extent similar to that achievable with the control cells (Figure 4c). To get a further understanding of RAP1 in mediating CP resistance, we performed a competition assay in which normal A549 cells (GFP^-^) were mixed with RAP1-overexpressing or -deleted cells (GFP^+^) at a 1 : 1 ratio before applied to CP treatment (Figure 4d). When treated with CP over time, the cells harboring overexpressed RAP1 became the dominant population while RAP1-deficient cells got rapidly eliminated (Figure 4d). These results indicate that RAP1 renders lung cancer cells resistant to CP treatment.

CP acts through generating DNA damage in the proliferative cancer cells, which ultimately leads to their apoptosis. However, RAP1 does not seem to have significant impact on CP induction of DNA damage *per se* (Supplementary Figures S6a and b). Hence, we questioned whether RAP1 acts on regulating the apoptotic status of CP-treated cells. Not surprisingly, an induction of cleaved caspase-3 was detected in A549 cells following CP treatment (Figure 4e). In the cells harboring overexpressed RAP1, despite a similar baseline extent of apoptosis, CP did not trigger an obvious upregulation of cleaved caspase-3; in contrast, CP facilitated the RAP1-deleted cells to express a high level of cleaved caspase-3 at an early time point during the treatment (Figure 4e). Similarly, the proportion of Annexin5^+^ apoptotic cells after CP treatment was decreased in RAP1-overexpressing cells and, conversely, increased in RAP1-deleted cells (Figure 4f). When examining the antiapoptosis factor BCL-2, we discovered a positive correlation between RAP1 and BCL-2 expression even without CP treatment (Figure 4e, comparing columns 1, 3 and 5). CP treatment slightly induced BCL-2 expression in cells transduced with control and RAP1-overexpression vectors, which might be a negative feedback of facilitated apoptosis (Figure 4e). However, little increase of BCL-2 was observed in CP-treated, RAP1-deleted cells (Figure 4e), suggesting that RAP1 is necessary for BCL-2 induction in response to CP. Thus we would conclude that RAP1 inhibits CP-induced apoptosis to mediate CP resistance.

### CP resistance is associated with RAP1-dependent NF-κB activation

**Figure 5:**
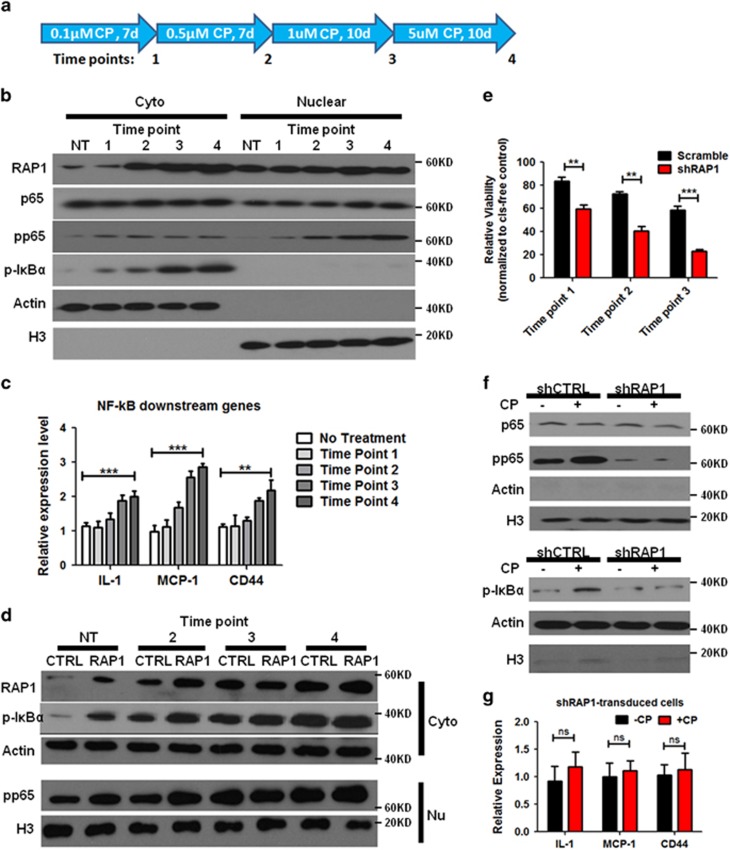
CP resistance is associated with RAP1-dependent NF-*κ*B signaling induction. (**a**) Schema of stepwise CP treatment on A549 cells. (**b** and **c**) RAP1 expression and NF-*κ*B activation during CP treatment. A549 cells treated with CP as depicted in panel (**a**) were harvested at the indicated time points; the protein expression of RAP1, p65, pp65 and p-I*κ*B*α* in the cytoplasmic and nuclear fractions was measured with western blotting analyses (**b**), representatives of three independent experiments; and the mRNA expression of NF-*κ*B signaling downstream factors was analyzed with qRT-PCR (**c**). (**d**) A549 cells with overexpressed RAP1 were treated with CP as depicted in panel **a**. RAP1, pp65 and p-I*κ*B*α* were detected in the cytoplasmic (Cyto) and nuclear (Nu) fractions of cell lysate at the indicated time points, representatives of three independent experiments. (**e**) Cell viability of A549 cells transduced with shRAP1 or scramble shRNA at different time points during the sequential CP treatment as depicted in panel **a**, normalized to the same type of cells cultured in CP-free media in respective time point. (**f**) Nuclear (upper panel) and cytoplasmic (lower panel) fractions were isolated from A549 cells transduced with shRAP1 or scramble shRNA and treated with 0.5 *μ*M of CP for 24 h and assayed for p65, pp65 and p-I*κ*B*α* protein levels, representatives of three independent experiments. (**g**) mRNA expression of NF-*κ*B signaling downstream factors in shRAP1-transduced A549 cells after 24 h culture in the presence or absence of 0.5 *μ*M of CP. Statistics were generated from three independent experiments. **P<0.01, ***P<0.001, Student’s *t*-test; error bar: ±S.D.

To further investigate the correlation between RAP1 expression and CP sensitivity, we treated A549 cells with increasing doses of CP to generate the cells bearing different extents of resistance (Figure 5a). Surviving cells were harvested at multiple time points to evaluate the RAP1 expression. Shown in Figure 5a, in the viable cells that sustain the escalating dosage of CP, cytoplasmic but not nuclear RAP1 expression was gradually induced, supporting our hypothesis that cytoplasmic RAP1 marks CP resistance. Moreover, similar induction was also observed when examining NF-*κ*B activity, as shown by changes in pp65 and p-I*κ*B*α* (Figure 5b). Notably, the increase of pp65 and p-I*κ*B*α* showed a delay when compared with RAP1 expression, suggesting their roles as the responders to RAP1 when encountering CP in the environment. Transcription of IL-1, MCP-1 and CD44 was also facilitated along the treatment process, which further demonstrated the activation of NF-*κ*B signaling (Figure 5c).

To clarify the requirement of RAP1 in activating NF-*κ*B signaling in response to CP, we further applied the sequential CP treatment to RAP1-overexpressing and -deleted cells. Consistent with the results depicted above, RAP1-overexpressing cells displayed an enhanced baseline NF-*κ*B activity, which was further strengthened by CP to a larger extent compared with the untransduced cells (Figure 5d). At the latest time point analyzed in this assay, however, there was no significant difference of NF-*κ*B activity between the RAP1-overexpressing and the untransduced cells (Figure 5d, columns 7 and 8), a result probably owing to the effect of continuous CP treatment in activating and saturating NF-*κ*B signaling. In contrast, 0.5 *μ*M of CP showed considerable toxicity to RAP1-deleted cells, and 1 *μ*M of CP eliminated almost all the cells (Figures 4a and 5e). More importantly, treatment using 0.1 and 0.5 *μ*M of CP did not induce NF-*κ*B signaling in the surviving RAP1-deleted cells, although considerate amount of cells still survived under these conditions (Figures 5f and g). Therefore, RAP1 is an intermediate mediator for CP-induced NF-*κ*B activation. Together, these results support the conclusion that RAP1 is necessary for the induction of NF-*κ*B in the cells surviving CP treatment. Further, given the observation by previous studies that NF-*κ*B signaling regulates BCL-2 transcription,^27^ the RAP1–NF-*κ*B–BCL2 axis is inferred in the lung cancer cells to induce CP resistance.

### RAP1 deletion is lethal to CP-resistant NSCLC cells

**Figure 6:**
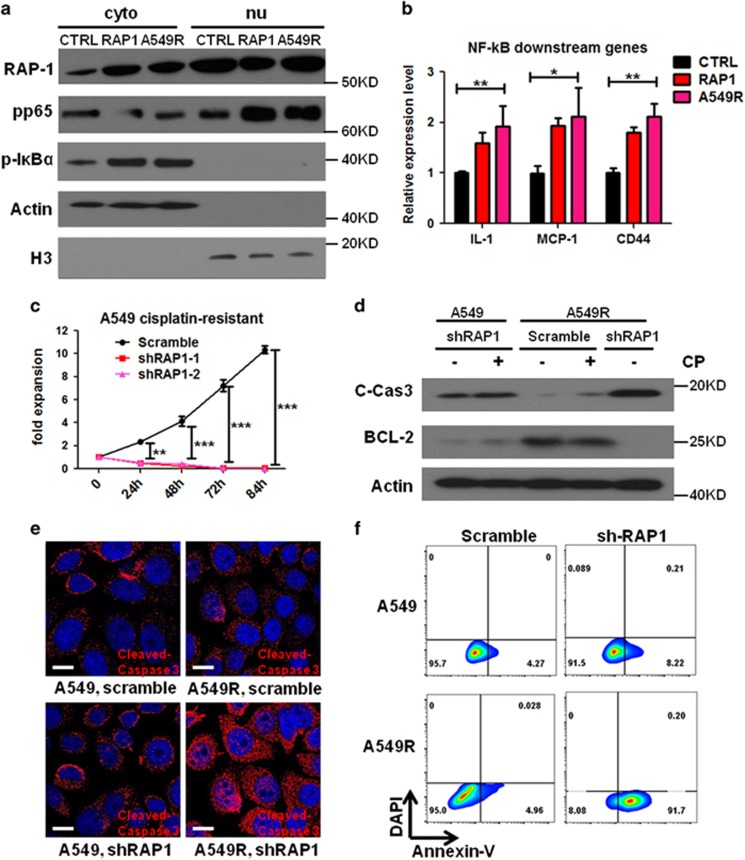
RAP1 deletion is lethal to CP-resistant NSCLC cells. (**a** and **b**) Protein expression of RAP1, pp65, p-I*κ*B*α* (**a**) and mRNA expression of NF-*κ*B signaling downstream factors (**b**) in control (CTRL), RAP1-overexpressed (RAP1) and CP-resistant (A549R) cells, representatives of three independent experiments. (**c**) Cell proliferation of A549R cells transduced with scramble shRNA or shRAP1 assayed with CCK-8 staining at the time point of transduction. Cells were precultured in CP-free media for 24 h before transduction. (**d**) RAP1-deleted A549 cells and scramble shRNA-transduced A549R cells were cultured in media containing 1 *μ*M CP (+) or CP-free media (−) for 24 h before being lysed to harvest protein. Lentiviral shRAP1 was introduced to A549R cells precultured in CP-free media for 24 h and protein harvested 12 h after transduction. Cleaved caspase-3 and BCL-2 were detected by western blotting. (**e**) IF assay of cleaved caspase-3 (red) in untreated A549 and A549R cells transduced with scramble shRNA or shRAP1. (**f**) Flow cytometric analysis for Annexin-V staining in A549 and A549R cells after RAP1 deletion. A549 cells were maintained in CP-free media and A549R cells were preconditioned in CP-free media for 24 h before analysis. Statistics were generated from three independent experiments. *P<0.05, **P<0.01, ***P<0.001, Student’s *t*-test; error bar: ±S.D.

Having demonstrated the role of RAP1 in inhibiting CP-induced apoptosis, we then aimed to harness RAP1 as a target to overcome CP resistance. As described above, RAP1 deletion increased the sensitivity of NSCLC cells to CP (Figure 5e), so we next evaluated the capability to target RAP1 in cells that have already established resistance. We generated CP-resistant A549 cells (A549R) using protocols described previously.^34, 35^ These A549R cells displayed upregulated cytoplasmic RAP1 and induced NF-*κ*B signaling (Figures 6a, b, Supplementary Figure S7b). The tolerance of these cells to CP was well characterized by treating with high dose of CP, which eliminated 60–90% of normal A549 cells within 24 h (Supplementary Figure S7a). The BCL-2 expression was much higher in A549R cells compared with parental A549 cells, probably contributed by the continuous CP treatment in maintaining resistance (Figure 6d and Supplementary Figure S7c). Inspired by the effect described above that RAP1 deletion sensitizes cells to CP treatment, we sought to attenuate CP resistance in A549R cells through deleting RAP1. Surprisingly, lethality was observed almost immediately after applying lentiviruses encoding sh-RAP1 to A549R cells (Figure 6c). To rule out the possibility that the CP used to maintain resistance might induce mortality, we cultured CP-resistant cells in CP-free media for 24 h before inducing RAP1 deletion. Such CP clearance did not affect the tolerance of the cells to CP, but RAP1-depleted cells still could not survive for 7 days (Figure 6c and Supplementary Figure S4d). Consistently, although scramble shRNA-transduced A549R cells had minor induction of apoptosis in response to CP treatment, an upregulation of cleaved caspase-3 and the almost complete loss of BCL-2 were observed in the RAP1-deleted A549R cells even though these cells had undergone CP-free culture prior to transduction (Figure 6d). Expression of cleaved caspase-3 in RAP1-deleted A549R cells was significantly higher than untreated RAP1-deleted A549 cells and similar to CP-treated RAP1-deleted A549 cells (Figures 6d and e). Therefore, RAP1 deletion alone is sufficient to cause an overwhelming lethality in the A549R cells through hyperactivation of apoptosis, for which further CP treatment is no longer required.

## Discussion

**Figure 7:**
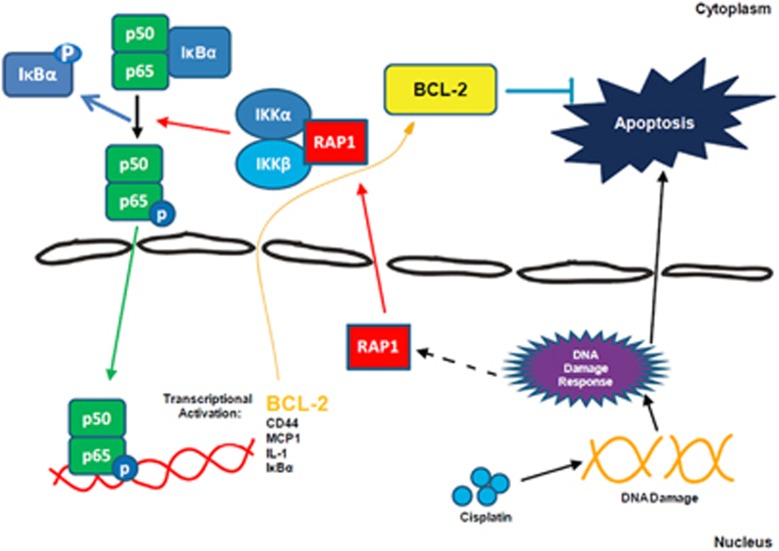
Schematic depiction of the proposed model. CP generates DNA damage, which eventually leads to cell apoptosis. Meanwhile, RAP1 is upregulated after CP treatment, possibly through a direct or indirect induction by DNA damage response. The cytoplasmic fraction of RAP1 thus acts to facilitate the IKK-mediated activation of NF-*κ*B signaling, which subsequently induces transcription of downstream factors, including the apoptosis inhibitor BCL-2. Therefore, RAP1 functions through activating NF-*κ*B signaling to mediate resistance to CP

Here we have identified an extratelomeric function of RAP1 acting through the NF-*κ*B signaling to promote lung cancer cell growth and mediate CP resistance (Figure 7). Our results show that cytoplasmic RAP1 is more enriched in human high-grade NSCLCs, which is agreed by previous observations in breast cancer.^17^ Several independent studies have confirmed that NSCLC cells acquired malignancy-associated phenotypes after repetitive exposure to the cytotoxic agent CP;^33, 36^ we demonstrate here that similar treatment also leads to the induction of RAP1 expression. The facilitating role of RAP1 in NSCLC progression is supported by our findings that shRNA-mediated RAP1 knockdown severely impairs the growth of lung adenocarcinoma and squamous carcinoma cell lines. One might argue that RAP1 deletion targets not only the cytoplasmic fraction but also the nuclear fraction. However, nuclear RAP1 has been demonstrated to be dispensable for maintaining chromosome integrity.^13^ Also, the p-I*κ*B*α* level was always associated with the RAP1 expression in this study, indicating that the main function of RAP1 in NSCLC cells comes from its cytoplasmic fraction.

Notably, while overexpressing RAP1 resulted in only a modest increase of cellular proliferation, it rendered the cells more resistant to CP treatment. Many studies have showed synergistic effects between CP and NF-*κ*B inhibitor but the induction of NF-*κ*B by CP seemed indirect.^31, 32^ CP acts directly on the double helix DNA to generate damage. As we observed only minor or no effect of RAP1 on CP induction of DNA damage in NSCLC cells (Supplementary Figure S6), we infer that RAP1 mediates CP-resistance through reducing cytotoxic response to the DNA damage induced by CP. We speculate that CP-induced DNA damage triggers the synthesis of shelterin-forming genes, including RAP1; RAP1 is not required for the chromatin protection but, when transported to the cytoplasm, acts as a mediator of CP resistance through activating NF-*κ*B signaling and subsequently inducing BCL-2 expression (Figure 7). Notably, we did observe a small fraction of RAP1-depleted cells that survived after CP treatment, pointing out the existence of redundant or additional mechanism that mediates CP resistance even in the absence of RAP1. However, those remaining cells failed to activate NF-*κ*B signaling even after CP exposure, demonstrating that NF-*κ*B induction is RAP1 dependent.

Interestingly, after RAP1 deletion, the transcription of I*κ*B*α*, which was both an inhibitor and a downstream target of NF-*κ*B,^17, 29^ was remarkably decreased. The total I*κ*B*α* protein, however, was only moderately decreased as a reduction of I*κ*B*α* phosphorylation will cause less I*κ*B*α* protein to be degraded. The synergistic effect we found between RAP1 deletion and TNF-*α* treatment (Supplementary Figure S8) could further prove that targeting RAP1 is able to inhibit NF-*κ*B activity, as TNF-*α* resistance is also correlated with NF-*κ*B activity.^37, 38, 39^ It is elucidated here that BCL-2 could be, at least one of, the downstream effectors of activated NF-*κ*B signaling contributing to CP resistance. Given the activity of NF-*κ*B on the promoter region of BCL-2 demonstrated by previous research,^26, 40^ RAP1 is most likely to mediate CP resistance through a transcriptional facilitation of the negative apoptosis regulator via NF-*κ*B. The RAP1–NF-*κ*B–BCL2 regulatory axis partially explains why cytoplasmic RAP1 is highly expressed in tissues of advanced NSCLCs and associated with poorer clinical outcome, although further analysis is needed to examine RAP1 expression in NSCLCs pre- and post-CP therapy. Moreover, as BCL-2 was demonstrated to in turn induce NF-*κ*B signaling,^41, 42^ it is thus possible that the mutual regulation between NF-*κ*B and BCL-2 could act as a positive feedback loop, providing an intriguing direction to investigate whether an intermediate increase in RAP1 is able to cause CP resistance similar to highly overexpressed RAP1.

We observed a very intriguing and potentially highly significant effect in the established CP-resistant NSCLC cells, where RAP1 deletion led to an almost complete elimination of the cells. It was hypothesized that the hyperactivated NF-*κ*B in these cells will generate large amount of TNF-*α*, leading to the induction of apoptosis when the NF-*κ*B signaling is disrupted by loss of RAP1. However, mortality still occurred when fresh media containing negligible amount of TNF-*α* was applied immediately after RAP1 deletion, suggesting that TNF-*α* is not the main reason for apoptosis induction. One possible explanation is that the survival of CP-resistant cells becomes more dependent on the RAP1–NF-*κ*B–BCL2 regulatory axis, making the deletion of any components of this pathway lethal to these cells. Under physiological conditions, cells respond to DNA damage by arresting the cell cycle, allowing extended time for the DNA repair machinery to enter the damage site to mediate appropriate correction of DNA structure.^43^ However, CP-resistant A549 cells do proliferate faster than normal ones, which actually makes the CP-induced DNA damage accumulate in these cells. One may also propose that the CP-induced DNA damage hastens the proapoptotic signal that is repressed by BCL-2 only when RAP1 is intact. Upon RAP1 deletion, the no longer suppressed proapoptotic force would thus lead the cells toward programmed cell death.

Further examination of DNA damage in CP-resistant cells are needed to test the hypotheses mentioned above, but it is clear that targeting RAP1 directs the future approach against the resistant subset of NSCLC cells after CP treatment. On the other hand, RAP1 deletion also sensitizes NSCLC cells to CP, opening up new methods to combine RAP1-targeting strategy and cytotoxic chemotherapy. Moreover, the expression of cytoplasmic RAP1 could serve as a biomarker to screen NSCLC patients for their sensitivity to CP, thus enabling the pre-CP application of chemotherapy agent to the cohort with better expected responses. Further, given the essential role of NF-*κ*B signaling in oncogenesis, there is also a great possibility that cytoplasmic RAP1 could be exploited as a diagnostic or therapeutic target in other types of cancer.

## Materials and methods

### Patient tissue and immunohistochemistry

Acquisition, paraffin embedding, antibody staining and pathological scoring of lung adenocarcinoma and squamous cell carcinoma samples were performed by Shanghai Outdo Biotech Company (151 Libing Road, Shanghai, China, 201213). All specimens were handed anonymously following the company’s protocol and ethical standards. Paraffin-embedded sections were immunolabeled with anti-Rap1 antibody (Cell Signaling Technology, Danvers, MA, USA #5433). Positive staining and staining scores were determined by a pathologist in cancer cells and peri-tumoral normal tissues. RAP1 expression was calculated by multiplying the percentage of positivity with staining score in each sample. Representative images of different staining scores are shown in Supplementary Figure S1e.

### Cell culture

A549, PC9, HCC827 and HSAEC1-KT cells were obtained from ATCC (Manassas, VA, USA) and CP-resistant cells were generated with the procedures as described previously.^34, 35^ The human bronchial epithelial cell line 16HBE was a kind gift from Sino-French Hoffman Institute, Guangzhou Medical University, Guangdong, China. All cells were cultured based on the guidance from the manufacturer. CP resistance was maintained at 5 *μ*M of CP during culture. All cells were grown as monolayer cultures and maintained in a humidified atmosphere of 5% CO_2_ in air at 37 °C. All the cells have been authenticated before use.

### Lentivirus transduction

Lentiviruses expressing RAP1, RAP1 shRNA or scramble shRNA were designed and generated by VectorBuilder (Santa Clara, CA, USA). Sequence of the construct is available upon request. Lentivirus transduction was performed in cell culture media as described above, and transduction efficiency was determined through GFP visualization through a DMI3000B microscope (Leica, Weztlar, Germany).

### Cell viability and colony formation analysis

Cell viability was analyzed by Cell Counting Kit 8 (Dojindo, Kumamoto, Japan) according to the manufacturer’s instructions and calculated as the percentages of control plated under the same conditions, based on the procedure of previous studies.^44, 45^ Cell proliferation was calculated as fold expansion in comparison to the viable cells plated. Colony formation was performed as described previously.^46^

### RNA extraction and qRT-PCR

For RNA extraction, cells were harvested, washed with PBS, homogenized in Trizol reagent (Life Technologies, Carlsbad, CA, USA) before proceeding to RNA isolation, by following the manufacturer’s instruction. Synthesis of cDNA was performed using the iScript cDNA Synthesis Kit (BIO-RAD, Hercules, CA, USA). Quantitative PCR was performed in a system of SsoFast EvaGreen Supermix (BIO-RAD) with the primers as following: GAPDH: 5′-ACAGTCCATGCCATCACTGCC-3′ (forward) and 5′-GCCTGCTTCACCACCTTCTTG-3′ (reverse); MCP-1: 5′-ACTGAAGCTCGTACTCTC-3′ (forward) and 5′-CTTGGGTTGTGGAGTGAG-3′ (reverse); CD44: 5′-CGGACACCATGGACAAGTTT-3′ (forward) and 5′-GAAAGCCTTGCAGAGGTCAG-3′ (reverse); IL-1: 5′-AGAAGCTTCCACCAATACTC-3′ (forward) and 5′-AGCACCTAGTTGTAAGGAAG-3′ (reverse); and I*κ*B*α*: 5′-CTCCGAGACTTTCGAGGAAATAC-3′ (forward) and 5′-GCCATTGTAGTTGGTAGCCTTCA-3′ (reverse). All expression was normalized to GAPDH.

### Western blotting analysis

Whole-cell lysate was generated with RIPA lysis and extraction buffer supplemented with protease inhibitors and phosphatase inhibitors (Thermo, Canoga Park, CA, USA); fractionated cell lysates were generated with NE-PER nuclear and cytoplasmic extraction reagents (Thermo). Electrophoresis and transfer were based on standard procedures as previously reported.^47^ Protein blots were probed with primary antibodies against Actin (Cell Signaling Technology #4970), Histone3 (Cell Signaling Technology #4499), RAP1 (Cell Signaling Technology #5433), p65 (Cell Signaling Technology #8242), p65-pSer536 (Cell Signaling Technology #3033), I*κ*B*α* (Cell Signaling Technology #4814), I*κ*B*α*-pSer32 (Cell Signaling Technology #2859), *γ*H2AX (Abcam, Cambridge, UK #11174), BCL-2 (Cell Signaling Technology #2870) and cleaved caspase-3 (Cell Signaling Technology #1658) followed by HRP-conjugated secondary antibodies (Cell Signaling Technology #7074 and #7076). Immunoblots were detected using ECL reagents (Thermo).

### Immunofluorescence

Cells were digested with trypsin and then plated for 24 h to adhere to chamber slides (Millipore, Billirica, MA, USA). Then the cells were fixed with 4% paraformaldehyde, permeabilized with 0.5% Triton-X and blocked with 2% of bovine serum albumin. Staining was performed using primary antibodies against IKK*α*, p65, pp65, BCL-2 I*κ*B*α*, p-I*κ*B*α*, cleaved caspase-3 (Cell Signaling Technology) same as above and *γ*H2AX (Abcam #11174) for 12–18 h followed by secondary staining using Alexa fluro594 goat-anti-rabbit or goat-anti-mouse IgG (Proteintech, Chicago, IL, USA) for 1 h and DAPI for 10 min. Fluorescence was detected via a TCS SP8 confocal microscope (Leica) and processed by Image J (NIH, Bethesda, MD, USA).

### Flow cytometry

Trypsinized cells were washed and resuspended in PBS supplemented with 2% FBS. For GFP detection, cells were directly proceeded to analysis. To examine apoptotic status, cells were stained with AnnexinV-PE (Beyotime, Shanghai, China). DAPI was added to all samples to exclude unviable cells no early than 1 min before analysis. Flow cytometric analysis was performed with Cytoflex (Beckman Coulter, Brea, CA, USA) and results processed via FlowJo v10 (Ashland, OR, USA).

### Statistics

Data analysis was performed using Prism v5.0 (GraphPad Software, La Jolla, CA, USA) and presented as stated in individual figure legends. Comparison was determined using Student’s *t*-test (two groups) or one-way ANOVA analysis with Sidak–Bonferroni correction (three or more groups).

## References

[bib1] Gridelli C, Rossi A, Carbone DP, Guarize J, Karachaliou N, Mok T et al. Non-small-cell lung cancer. Nat Rev Dis Primers 2015; 1: 15009. 27188576 10.1038/nrdp.2015.9

[bib2] Schaake-Koning C, van den Bogaert W, Dalesio O, Festen J, Hoogenhout J, van Houtte P et al. Effects of concomitant Cisplatin and radiotherapy on inoperable non-small-cell lung cancer. N Engl J Med 1992; 326: 524–530. 1310160 10.1056/NEJM199202203260805

[bib3] Olaussen KA, Dunant A, Fouret P, Brambilla E, Andre F, Haddad V et al. DNA repair by ERCC1 in non-small-cell lung cancer and Cisplatin-based adjuvant chemotherapy. N Engl J Med 2006; 355: 983–991. 16957145 10.1056/NEJMoa060570

[bib4] Mitsudomi T, Morita S, Yatabe Y, Negoro S, Okamoto I, Tsurutani J et al. Gefitinib versus Cisplatin plus docetaxel in patients with non-small-cell lung cancer harbouring mutations of the epidermal growth factor receptor (WJTOG3405): an open label, randomised phase 3 trial. Lancet Oncol 2010; 11: 121–128. 20022809 10.1016/S1470-2045(09)70364-X

[bib5] Scagliotti GV, Parikh P, von Pawel J, Biesma B, Vansteenkiste J, Manegold C et al. Phase III study comparing Cisplatin plus gemcitabine with Cisplatin plus pemetrexed in chemotherapy-naive patients with advanced-stage non-small-cell lung cancer. J Clin Oncol 2008; 26: 3543–3551. 18506025 10.1200/JCO.2007.15.0375

[bib6] Martinez P, Blasco MA. Telomeric and extra-telomeric roles for telomerase and the telomere-binding proteins. Nat Rev Cancer 2011; 11: 161–176. 21346783 10.1038/nrc3025

[bib7] Li B, Oestreich S, de Lange T. Identification of human Rap1: implications for telomere evolution. Cell 2000; 101: 471–483. 10850490 10.1016/s0092-8674(00)80858-2

[bib8] Shore D, Nasmyth K. Purification and cloning of a DNA binding protein from yeast that binds to both silencer and activator elements. Cell 1987; 51: 721–732. 3315231 10.1016/0092-8674(87)90095-x

[bib9] Braig M, Pallmann N, Preukschas M, Steinemann D, Hofmann W, Gompf A et al. A 'telomere-associated secretory phenotype' cooperates with BCR-ABL to drive malignant proliferation of leukemic cells. Leukemia 2014; 28: 2028–2039. 24603533 10.1038/leu.2014.95

[bib10] Aoude LG, Pritchard AL, Robles-Espinoza CD, Wadt K, Harland M, Choi J et al. Nonsense mutations in the shelterin complex genes ACD and TERF2IP in familial melanoma. J Natl Cancer Inst 2015; 107: dju408. 10.1093/jnci/dju408 PMC4334787 25505254

[bib11] Massion PP, Zou Y, Chen H, Jiang A, Coulson P, Amos CI et al. Smoking-related genomic signatures in non-small cell lung cancer. Am J Respir Crit Care Med 2008; 178: 1164–1172. 18776155 10.1164/rccm.200801-142OC PMC2720147

[bib12] Sweet-Cordero A, Tseng GC, You H, Douglass M, Huey B, Albertson D et al. Comparison of gene expression and DNA copy number changes in a murine model of lung cancer. Genes Chromosomes Cancer 2006; 45: 338–348. 16323170 10.1002/gcc.20296

[bib13] Kabir S, Hockemeyer D, de Lange T. TALEN gene knockouts reveal no requirement for the conserved human shelterin protein Rap1 in telomere protection and length regulation. Cell Rep 2014; 9: 1273–1280. 25453752 10.1016/j.celrep.2014.10.014 PMC4254571

[bib14] Sfeir A, Kabir S, van Overbeek M, Celli GB, de Lange T. Loss of Rap1 induces telomere recombination in the absence of NHEJ or a DNA damage signal. Science 2010; 327: 1657–1661. 20339076 10.1126/science.1185100 PMC2864730

[bib15] Yeung F, Ramirez CM, Mateos-Gomez PA, Pinzaru A, Ceccarini G, Kabir S et al. Nontelomeric role for Rap1 in regulating metabolism and protecting against obesity. Cell Rep 2013; 3: 1847–1856. 23791522 10.1016/j.celrep.2013.05.032 PMC5523811

[bib16] Martinez P, Thanasoula M, Carlos AR, Gomez-Lopez G, Tejera AM, Schoeftner S et al. Mammalian Rap1 controls telomere function and gene expression through binding to telomeric and extratelomeric sites. Nat Cell Biol 2010; 12: 768–780. 20622869 10.1038/ncb2081 PMC3792482

[bib17] Teo H, Ghosh S, Luesch H, Ghosh A, Wong ET, Malik N et al. Telomere-independent Rap1 is an IKK adaptor and regulates NF-kappaB-dependent gene expression. Nat Cell Biol 2010; 12: 758–767. 20622870 10.1038/ncb2080

[bib18] Tang X, Liu D, Shishodia S, Ozburn N, Behrens C, Lee JJ et al. Nuclear factor-kappaB (NF-kappaB) is frequently expressed in lung cancer and preneoplastic lesions. Cancer 2006; 107: 2637–2646. 17078054 10.1002/cncr.22315

[bib19] Kelliher MA, Grimm S, Ishida Y, Kuo F, Stanger BZ, Leder P. The death domain kinase RIP mediates the TNF-induced NF-kappaB signal. Immunity 1998; 8: 297–303. 9529147 10.1016/s1074-7613(00)80535-x

[bib20] Tanaka K, Babic I, Nathanson D, Akhavan D, Guo D, Gini B et al. Oncogenic EGFR signaling activates an mTORC2-NF-kappaB pathway that promotes chemotherapy resistance. Cancer Discov 2011; 1: 524–538. 22145100 10.1158/2159-8290.CD-11-0124 PMC3229221

[bib21] Prasad S, Ravindran J, Aggarwal BB. NF-kappaB and cancer: how intimate is this relationship. Mol Cell Biochem 2010; 336: 25–37. 19823771 10.1007/s11010-009-0267-2 PMC3148942

[bib22] Jones DR, Broad RM, Madrid LV, Baldwin AS Jr, Mayo MW. Inhibition of NF-kappaB sensitizes non-small cell lung cancer cells to chemotherapy-induced apoptosis. Ann Thorac Surg 2000; 70: 930–936 discussion 936–937. 11016336 10.1016/s0003-4975(00)01635-0

[bib23] Arlt A, Gehrz A, Muerkoster S, Vorndamm J, Kruse ML, Folsch UR et al. Role of NF-kappaB and Akt/PI3K in the resistance of pancreatic carcinoma cell lines against gemcitabine-induced cell death. Oncogene 2003; 22: 3243–3251. 12761494 10.1038/sj.onc.1206390

[bib24] Wang SJ, Gao Y, Chen H, Kong R, Jiang HC, Pan SH et al. Dihydroartemisinin inactivates NF-kappaB and potentiates the anti-tumor effect of gemcitabine on pancreatic cancer both *in vitro* and *in vivo*. Cancer Lett 2010; 293: 99–108. 20137856 10.1016/j.canlet.2010.01.001

[bib25] Wang Y, Wang X, Zhao H, Liang B, Du Q. Clusterin confers resistance to TNF-alpha-induced apoptosis in breast cancer cells through NF-kappaB activation and Bcl-2 overexpression. J Chemother 2012; 24: 348–357. 23174100 10.1179/1973947812Y.0000000049

[bib26] Viatour P, Bentires-Alj M, Chariot A, Deregowski V, de Leval L, Merville MP et al. NF- kappa B2/p100 induces Bcl-2 expression. Leukemia 2003; 17: 1349–1356. 12835724 10.1038/sj.leu.2402982

[bib27] Heckman CA, Mehew JW, Boxer LM. NF-kappaB activates Bcl-2 expression in t(14;18) lymphoma cells. Oncogene 2002; 21: 3898–3908. 12032828 10.1038/sj.onc.1205483

[bib28] Fahy BN, Schlieman MG, Mortenson MM, Virudachalam S, Bold RJ. Targeting BCL-2 overexpression in various human malignancies through NF-kappaB inhibition by the proteasome inhibitor bortezomib. Cancer Chemother Pharmacol 2005; 56: 46–54. 15791457 10.1007/s00280-004-0944-5

[bib29] Storci G, Sansone P, Mari S, D'Uva G, Tavolari S, Guarnieri T et al. TNFalpha up-regulates SLUG via the NF-kappaB/HIF1alpha axis, which imparts breast cancer cells with a stem cell-like phenotype. J Cell Physiol 2010; 225: 682–691. 20509143 10.1002/jcp.22264 PMC2939957

[bib30] Kelly PN, Strasser A. The role of Bcl-2 and its pro-survival relatives in tumourigenesis and cancer therapy. Cell Death Differ 2011; 18: 1414–1424. 21415859 10.1038/cdd.2011.17 PMC3149740

[bib31] Venkatraman M, Anto RJ, Nair A, Varghese M, Karunagaran D. Biological and chemical inhibitors of NF-kappaB sensitize SiHa cells to Cisplatin-induced apoptosis. Mol Carcinog 2005; 44: 51–59. 16044419 10.1002/mc.20116

[bib32] Hernandez-Flores G, Ortiz-Lazareno PC, Lerma-Diaz JM, Dominguez-Rodriguez JR, Jave-Suarez LF, Aguilar-Lemarroy Adel C et al. Pentoxifylline sensitizes human cervical tumor cells to Cisplatin-induced apoptosis by suppressing NF-kappa B and decreased cell senescence. BMC Cancer 2011; 11: 483. 22074157 10.1186/1471-2407-11-483 PMC3229613

[bib33] Barr MP, Gray SG, Hoffmann AC, Hilger RA, Thomale J, O'Flaherty JD et al. Generation and characterisation of Cisplatin-resistant non-small cell lung cancer cell lines displaying a stem-like signature. PLoS ONE 2013; 8: e54193. 23349823 10.1371/journal.pone.0054193 PMC3547914

[bib34] Hong WS, Saijo N, Sasaki Y, Minato K, Nakano H, Nakagawa K et al. Establishment and characterization of Cisplatin-resistant sublines of human lung cancer cell lines. Int J Cancer 1988; 41: 462–467. 3346112 10.1002/ijc.2910410325

[bib35] Zhou B, Huang J, Zuo Y, Li B, Guo Q, Cui B et al. 2a, a novel curcumin analog, sensitizes Cisplatin-resistant A549 cells to Cisplatin by inhibiting thioredoxin reductase concomitant oxidative stress damage. Eur J Pharmacol 2013; 707: 130–139. 23524096 10.1016/j.ejphar.2013.03.014

[bib36] Yang Y, Li H, Hou S, Hu B, Liu J, Wang J. The noncoding RNA expression profile and the effect of lncRNA AK126698 on Cisplatin resistance in non-small-cell lung cancer cell. PLoS ONE 2013; 8: e65309. 23741487 10.1371/journal.pone.0065309 PMC3669360

[bib37] Cheah SC, Appleton DR, Lee ST, Lam ML, Hadi AHA, Mustafa MR et al. Inhibits the growth of A549 cells through induction of apoptosis and inhibition of NF-KappaB translocation. Molecules 2011; 16: 2583–2598. 21441862 10.3390/molecules16032583 PMC6259648

[bib38] Lee KY, Park JS, Jee YK, Rosen GD. Triptolide sensitizes lung cancer cells to TNF-related apoptosis-inducing ligand (TRAIL)-induced apoptosis by inhibition of NF-kappaB activation. Exp Mol Med 2002; 34: 462–468. 12526088 10.1038/emm.2002.64

[bib39] Aggarwal BB. Signalling pathways of the TNF superfamily: a double-edged sword. Nat Rev Immunol 2003; 3: 745–756. 12949498 10.1038/nri1184

[bib40] De Smaele E, Zazzeroni F, Papa S, Nguyen DU, Jin R, Jones J et al. Induction of gadd45beta by NF-kappaB downregulates pro-apoptotic JNK signalling. Nature 2001; 414: 308–313. 11713530 10.1038/35104560

[bib41] Kirshenbaum LA. Bcl-2 intersects the NFkappaB signalling pathway and suppresses apoptosis in ventricular myocytes. Clin Invest Med 2000; 23: 322–330. 11055326

[bib42] Grimm S, Bauer MK, Baeuerle PA, Schulze-Osthoff K. Bcl-2 down-regulates the activity of transcription factor NF-kappaB induced upon apoptosis. J Cell Biol 1996; 134: 13–23. 8698809 10.1083/jcb.134.1.13 PMC2120920

[bib43] Pearl LH, Schierz AC, Ward SE, Al-Lazikani B, Pearl FMG. Therapeutic opportunities within the DNA damage response. Nat Rev Cancer 2015; 15: 166–180. 25709118 10.1038/nrc3891

[bib44] Chen Y, Meng D, Wang H, Sun R, Wang D, Wang S et al. VAMP8 facilitates cellular proliferation and temozolomide resistance in human glioma cells. Neuro Oncol 2015; 17: 407–418. 25209430 10.1093/neuonc/nou219 PMC4483099

[bib45] Fan J, Zeng X, Li Y, Wang S, Wang Z, Sun Y et al. Autophagy plays a critical role in ChLym-1-induced cytotoxicity of non-hodgkin's lymphoma cells. PLoS ONE 2013; 8: e72478. 24015249 10.1371/journal.pone.0072478 PMC3756084

[bib46] Wang H, Zhang SY, Wang S, Lu J, Wu W, Weng L et al. REV3L confers chemoresistance to Cisplatin in human gliomas: the potential of its RNAi for synergistic therapy. Neuro Oncol 2009; 11: 790–802. 19289490 10.1215/15228517-2009-015 PMC2802399

[bib47] Li XF, Liu ST, Huang HB, Liu NN, Zhao C, Liao SY et al. Gambogic acid is a tissue-specific proteasome inhibitor *in vitro* and *in vivo*. Cell Rep 2013; 3: 211–222. 23260670 10.1016/j.celrep.2012.11.023

