# Supplementary material for: Cytoplasmic RAP1 mediates cisplatin resistance of non-small cell lung cancer
Source: Cell Death Dis. 2017 May 18;8(5):e2803–. doi: 10.1038/cddis.2017.210 (PMC5520727; doi:10.1038/cddis.2017.210)
Supplement: Supplementary Table S1 [file cddis2017210x1.docx]

**Supplemental Table Legends**

**Supplemental Table1 RAP1 expression on lung adenocarcinoma tissues.** RAP1 expression is determined by immunohistochemistry on tissues (tumor and peri-tumoral normal tissues) from lung adenocarcinoma patients. Information of tumor stages and patient survival are included. NA: not applicable (due to unavailable peri-tumoral tissues or unsuccessful staining)

**Supplemental Table2 RAP1 expression on lung squamous cell carcinoma tissues.** RAP1 expression is determined by immunohistochemistry on tissues (tumor and peri-tumoral normal tissues) from lung squamous cell carcinoma patients. Information of tumor stages are included. NA: not applicable (due to unavailable peri-tumoral tissues or unsuccessful staining)
