# Supplementary figures and images for: Cytoplasmic RAP1 mediates cisplatin resistance of non-small cell lung cancer
Source: Cell Death Dis. 2017 May 18;8(5):e2803–. doi: 10.1038/cddis.2017.210 (PMC5520727; doi:10.1038/cddis.2017.210)

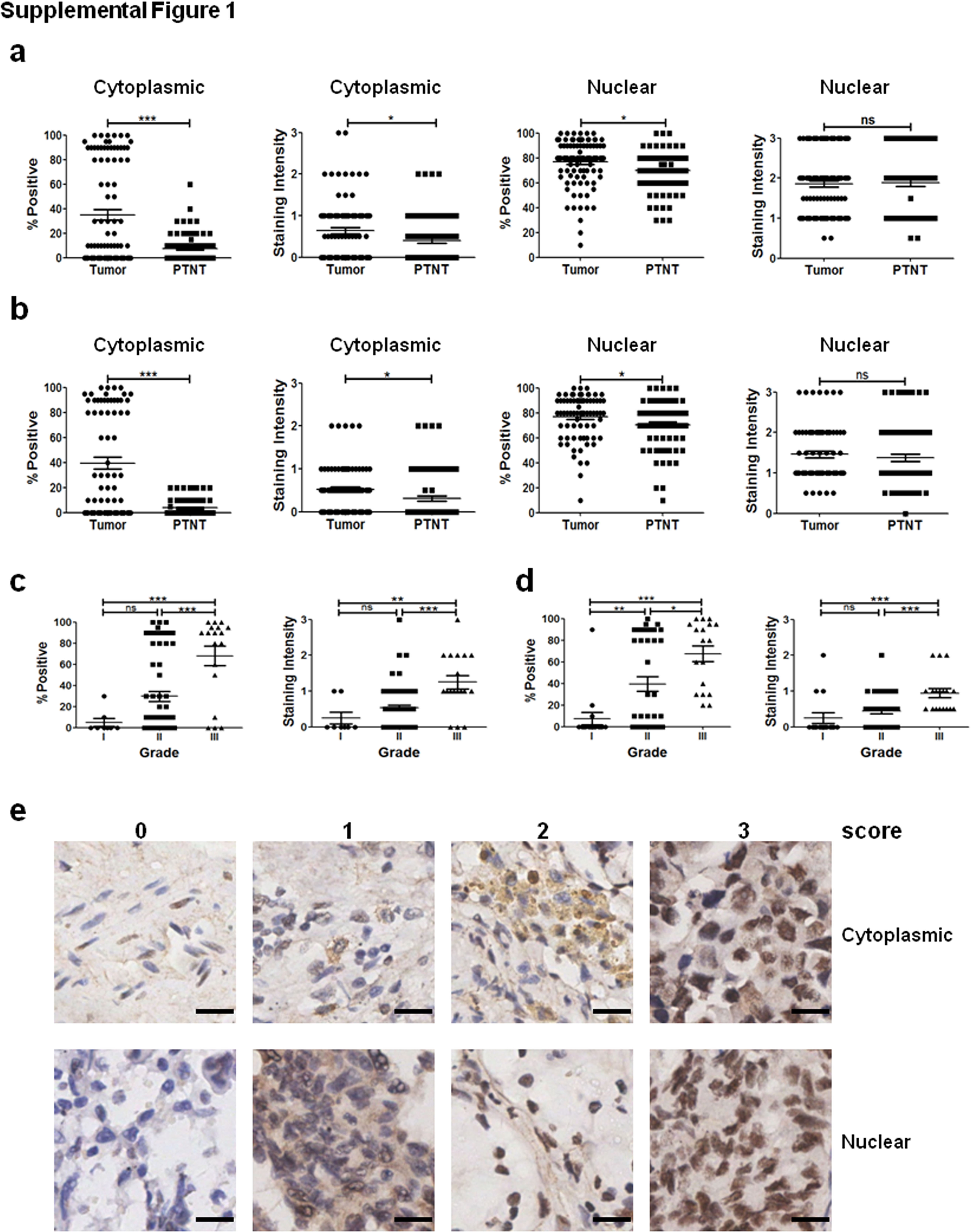

Supplement: Supplementary Figure S1 [file cddis2017210x4.tif]

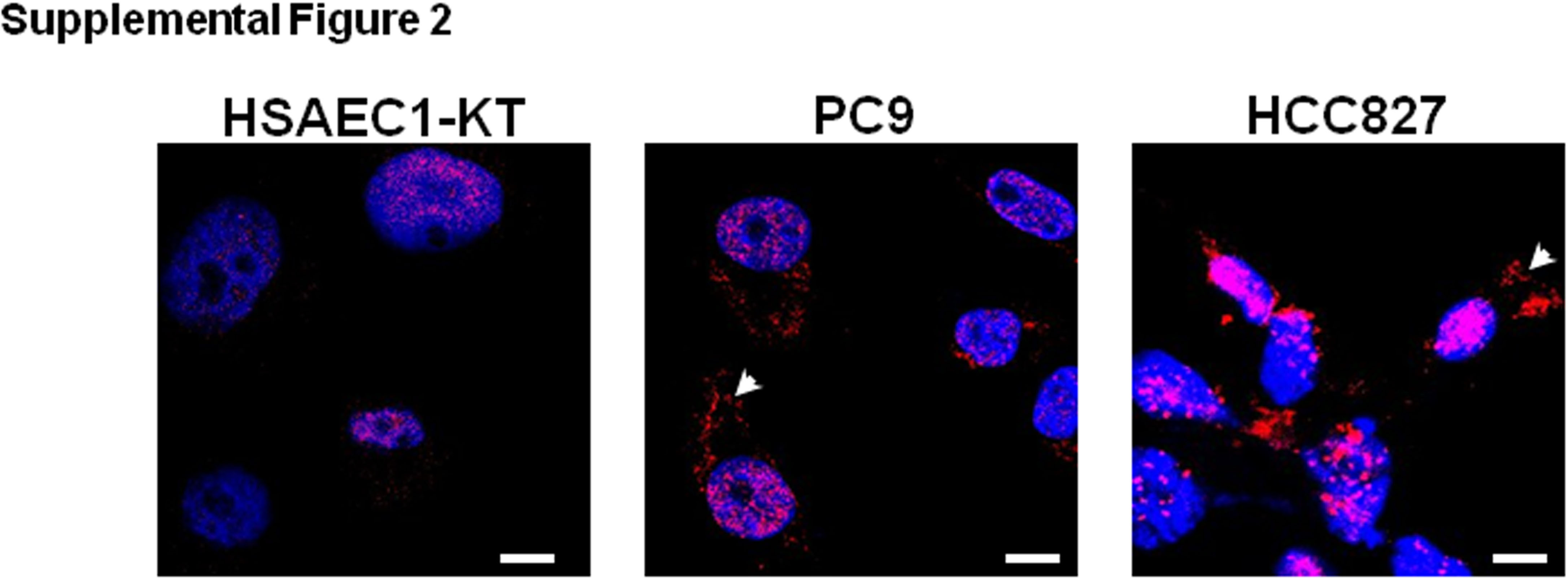

Supplement: Supplementary Figure 2 [file cddis2017210x5.tif]

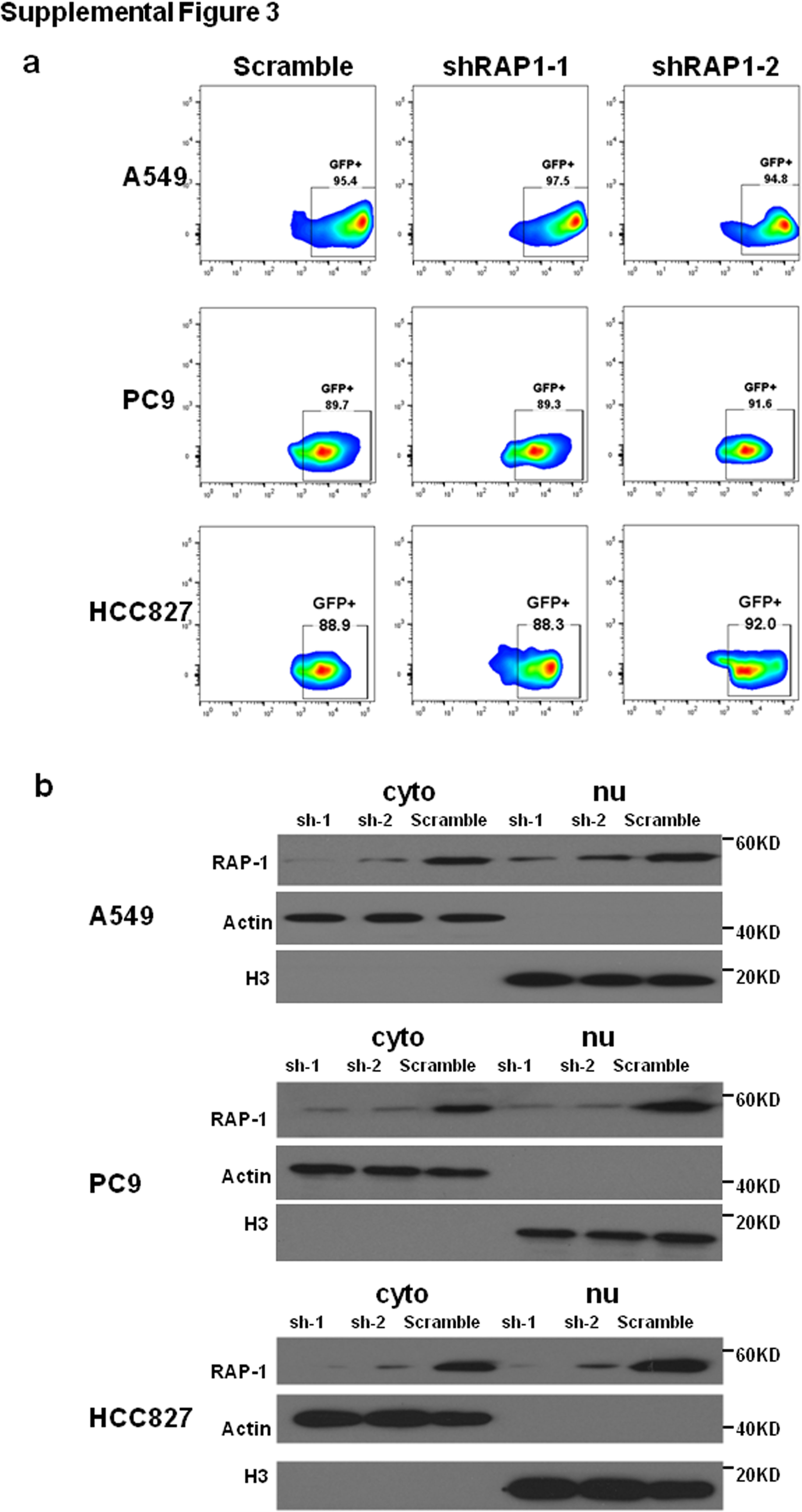

Supplement: Supplementary Figure 3 [file cddis2017210x6.tif]

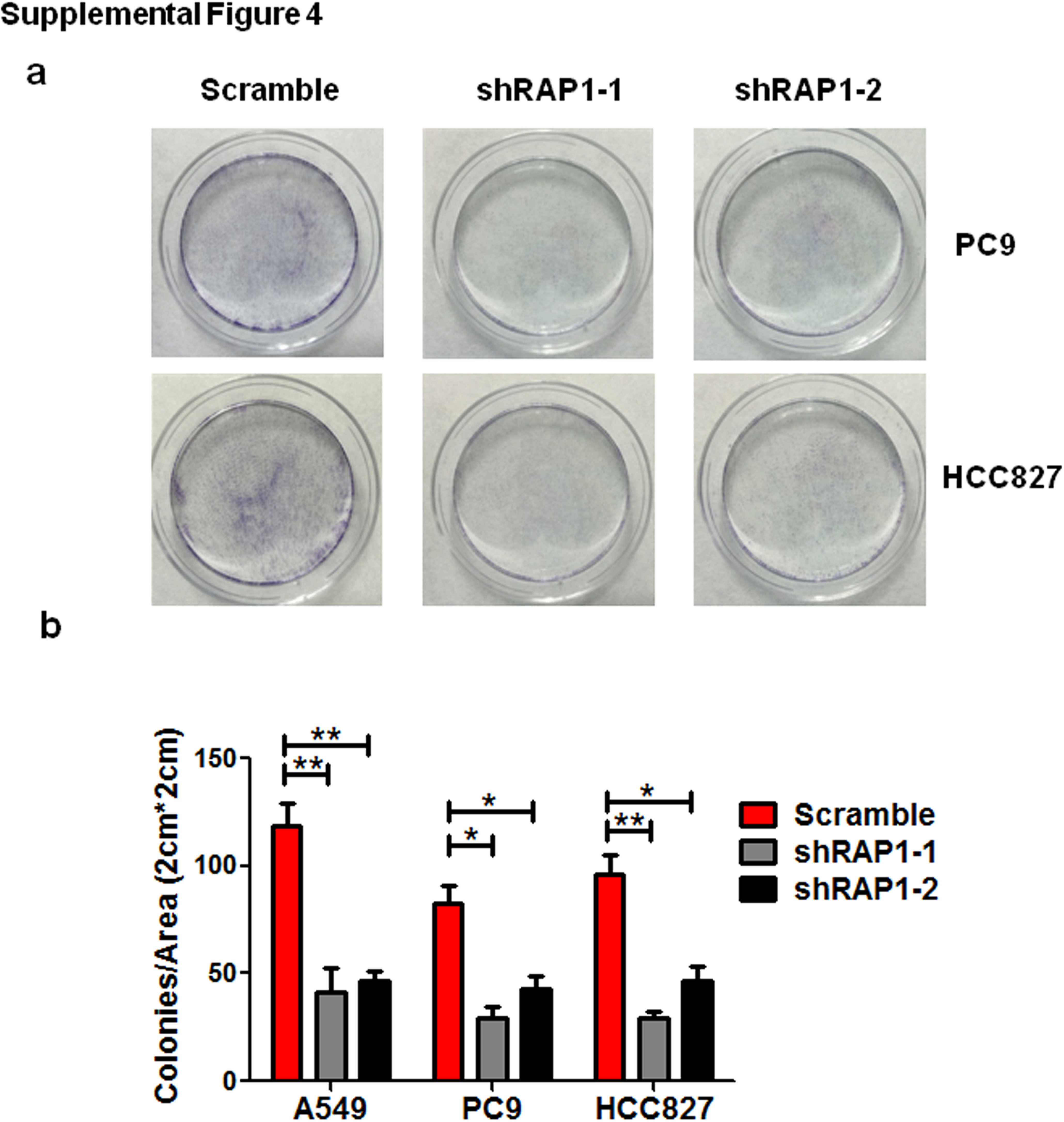

Supplement: Supplementary Figure 4 [file cddis2017210x7.tif]

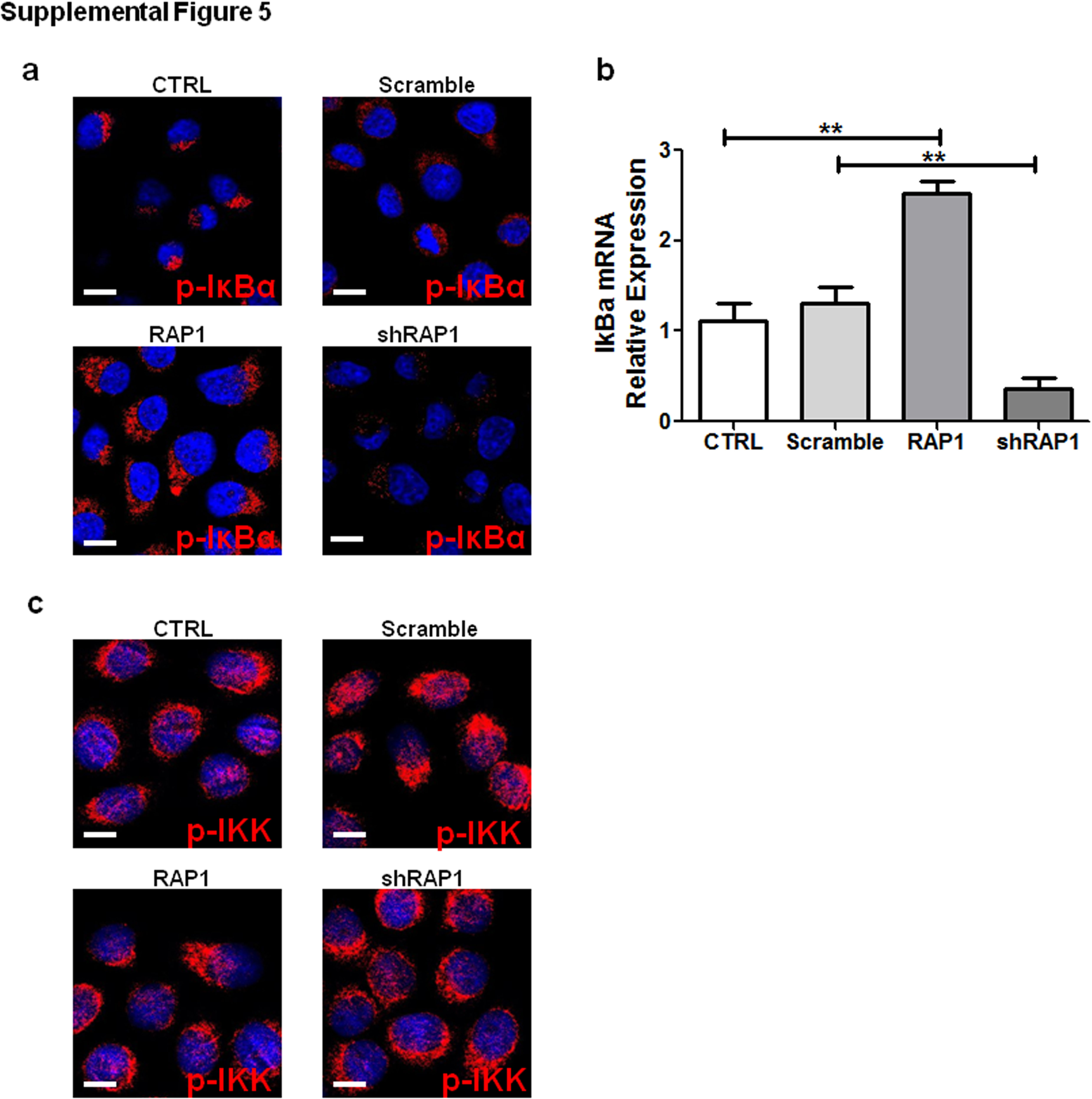

Supplement: Supplementary Figure 5 [file cddis2017210x8.tif]

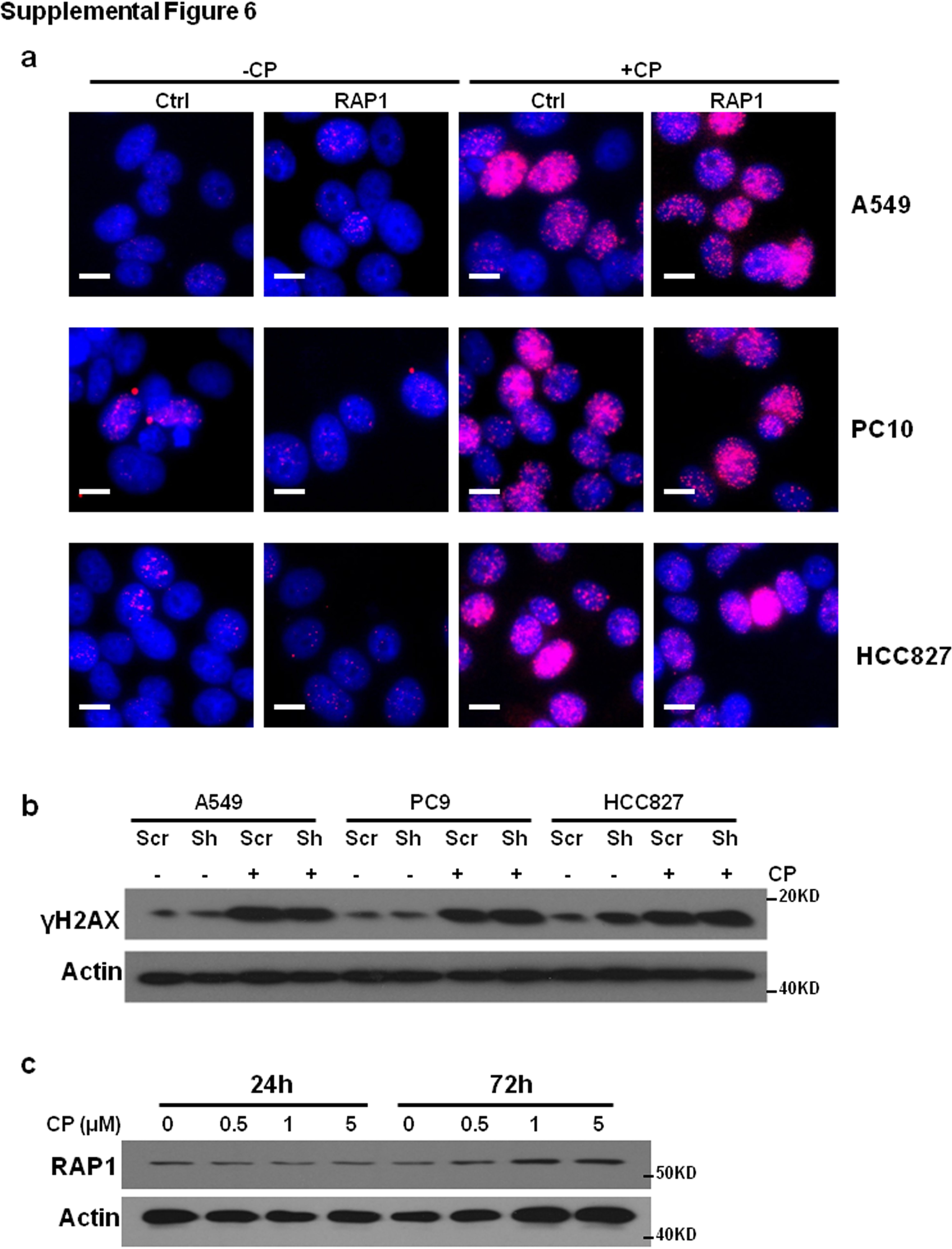

Supplement: Supplementary Figure 6 [file cddis2017210x9.tif]

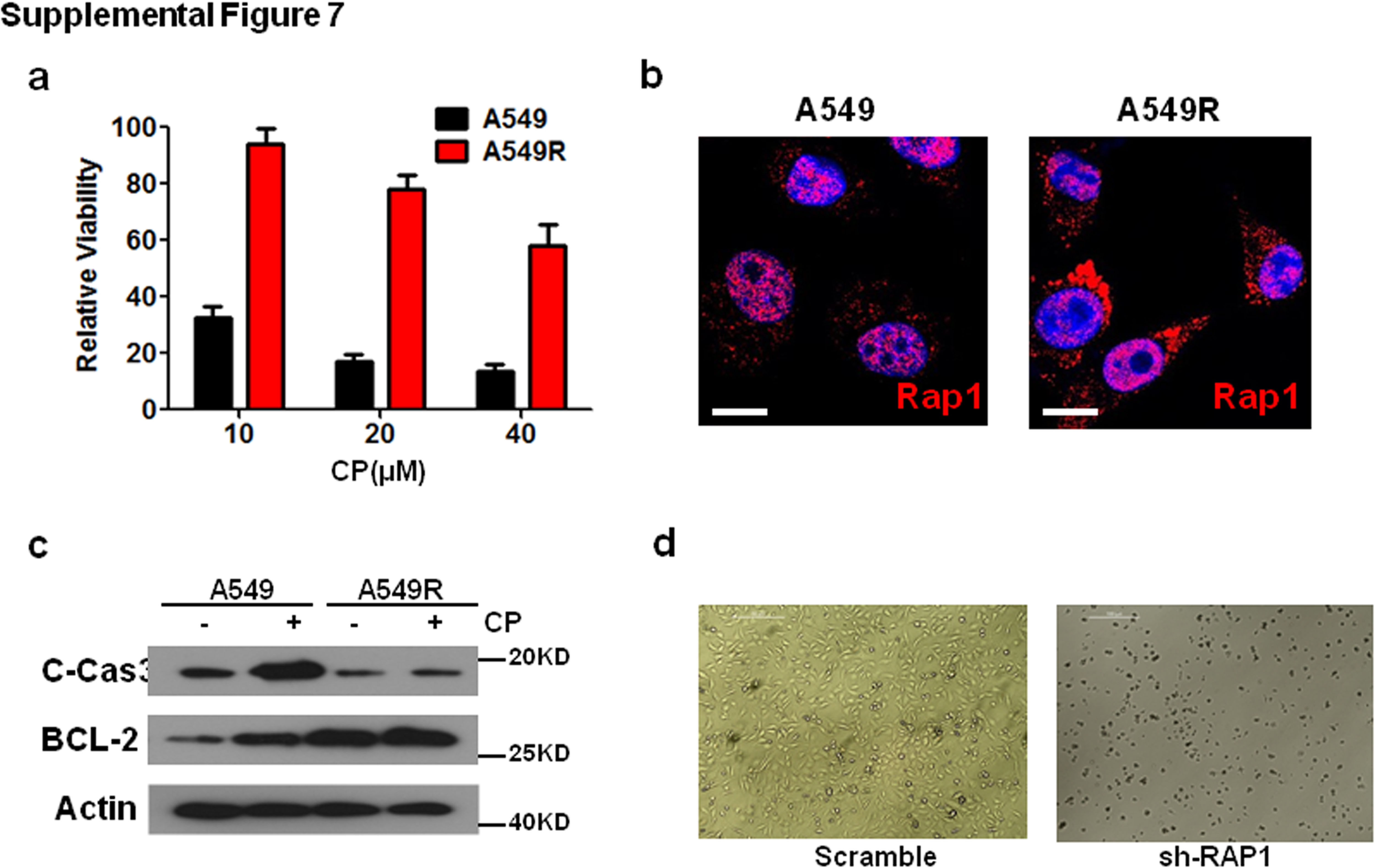

Supplement: Supplementary Figure 7 [file cddis2017210x10.tif]

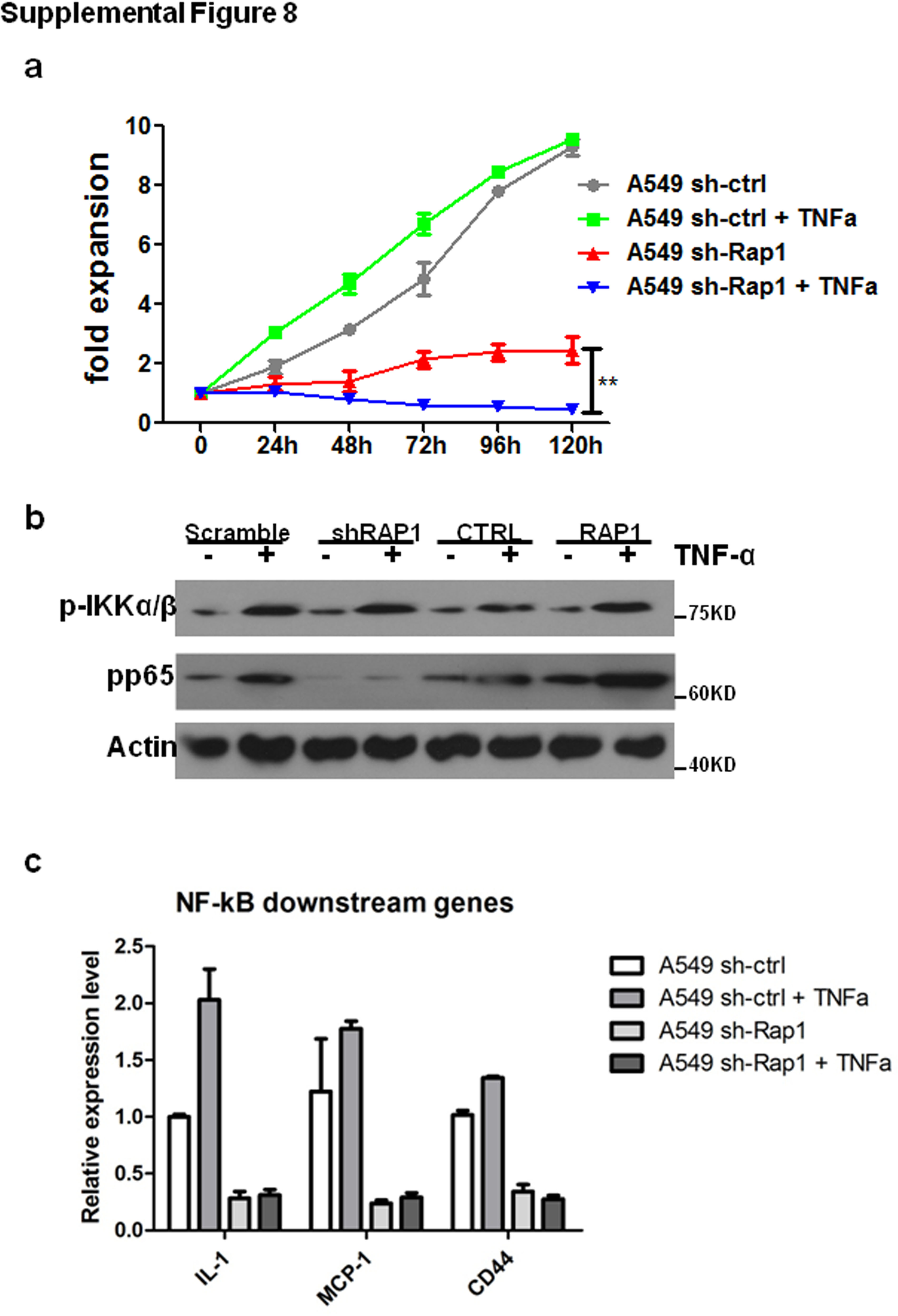

Supplement: Supplementary Figure 8 [file cddis2017210x11.tif]
